# ‘Mental health was always the last; always an afterthought’: policy analysis of domestic financing and prioritisation for mental health in Ghana

**DOI:** 10.1192/bjo.2026.10984

**Published:** 2026-07-14

**Authors:** Leonard Baatiema, Leveana Gyimah, Joana Ansong, Dan Chisholm, Bruno Meessen

**Affiliations:** Department of Health Policy, Planning and Management, School of Public Health, University of Ghana, Accra, Ghana; Department of Global Health and Population, Harvard T.H. Chan School of Public Health, Boston, Massachusetts, USA; Ghana Country Office, World Health Organization, Accra, Ghana; Department of Noncommunicable Diseases and Mental Health, World Health Organization, Geneva, Switzerland; Department of Performance, Financing and Delivery, World Health Organization, Geneva, Switzerland

**Keywords:** Mental health, priority-setting, agenda setting, financing, Ghana

## Abstract

**Background:**

Mental health remains a low health priority globally. Despite the high burden of mental health in Ghana, little is known about the extent of financing and prioritisation by policymakers and other relevant stakeholders.

**Aims:**

This study aims to understand the determinants of low domestic public financing and the prioritisation of mental health in Ghana, and to identify opportunities and strategies to increase policy attention.

**Method:**

Kingdon’s multiple streams framework was used to map barriers to domestic financing for mental health. We conducted a document analysis of relevant policies, strategies and articles, and carried out semi-structured interviews with relevant national stakeholders. We analysed interview transcripts deductively following the key domains for the multiple streams framework.

**Results:**

We found that low public financing for mental health stems from several issues at the level of the ‘problem stream’: low awareness and misunderstandings in general society, stigma and discrimination, insufficient public funding of the health sector in general, catastrophic healthcare expenditure insufficiently documented, limited political attention to mental health and limited donor interest. There is clear evidence of dynamism on the ‘policy solution stream’, including development of national laws, policies, strategies and institutions; adoption of international strategies and recommendations; and existence of policy entrepreneurs and windows. The democratic system of Ghana creates opportunities in the ‘politics stream’, but also raises the issue of continuity across governments.

**Conclusions:**

In Ghana, mental health is not yet sufficiently recognised as a societal issue, which affects demand for services and contributes to the low policy priority and resource allocation by the government. Greater investment is therefore needed to raise public awareness through coalition building, research, campaigns and education activities.

Worldwide, evidence suggests that mental health conditions are a leading cause of the global disease burden and have increased significantly in all countries.^1^ In 2019, mental disorders accounted for approximately 4.9% of global disability-adjusted life-years lost,^2^ and constituted 17.2% of total years lived in disability globally in 2021.^3^ This rise in the global burden of mental health is attributable to major demographic, environmental and societal transitions. This also applies to Africa, where the population grew by 49% but the number of years lost to disability attributed to mental and substance use disorders increased by about 52% from 2000 to 2015.^4^ Besides the related morbidity and mortality, mental health conditions raise extra societal problems; for instance, it is reported that the quality of life of people with mental health conditions is further exacerbated by institutionalised stigmatisation and discrimination.^5,6^

A critical issue of global concern is also the accessibility of quality care for people living with mental health conditions. Access is variably marked across and within regions and countries, with poor and vulnerable people experiencing exacerbated access challenges.^7^ Further, compared with physical health, the quality of mental health services is markedly suboptimal.^8^ Despite the high burden of limited access to quality mental health services, the general pattern is low political and policy interest in mental health, characterised by low prioritisation and resource allocation.^8,9^ An earlier study reported that globally, on average, only 2% of government budgets for health are allocated to mental healthcare, and treatment of mental health cases is rarely included in national health insurance or reimbursement schemes.^10^ In a sample of 11 African countries, spending on mental health was, on average, about 1% of total health budgets, ranging from 0.022% in Niger in 2015, to 4.7% in Gabon in 2016.^11^

In Ghana, mental health conditions continue to rise. A recent report estimated that about 2.6 million people in Ghana live with some form of mental health condition, representing 10.7% of the country’s population.^1^ Despite this situation, there is a disconnect between the vast mental health burden and the national response and investment to tackle this burden; the health system’s response to addressing the population’s needs remains poor, with previous studies highlighting gaps in mental healthcare,^12,13^ with treatment gaps estimated to be about 95–98%.^12^ Despite this, funding allocation to mental health remains low. The annual government expenditure on mental health was 0.05% in 2020, 0.07% in 2021 and 0.1% in 2022.^14^

As part of global efforts to galvanise support and mobilise resources for mental health, a Global Dialogue Meeting on the financing of non-communicable diseases (NCDs) and mental health took place in Washington, DC, USA, in June 2024.^9^ The World Health Organization (WHO) and the World Bank commissioned studies about the enablers and barriers to financing and prioritising NCDs and mental health in selected countries to inform this dialogue. Ghana was one of the three countries in which primary data collection was conducted. In this paper, we report findings specific to mental health (findings related to NCDs have been reported elsewhere).^15^

## Study objective and conceptual framework

The main research question concerns the reasons for the relatively low allocation of public financial resources to mental health in Ghana. To structure our investigation, we used Kingdon’s multiple streams framework (MSF),^16^ which focuses on the dynamics of agenda setting and policy prioritisation. The Kingdon framework has been widely applied across contexts and to various health policy issues,^17,18^ including by us to the specific case of prioritising diabetes and other NCDs in Ghana.^15^

According to Kingdon, three ‘streams’ influence policy development. They must converge for a policy issue to be prioritised by governments and to make its way onto the agenda-setting and business of government. The ‘problem stream’ describes how stakeholders, the media and the public understand or consider the issue as a significant societal problem necessitating prioritisation or policy action. The ‘policy solution stream’ refers to the existing actions developed by ‘policy entrepreneurs’, such as think tanks, researchers, activists, lobby groups or even private citizens, in putting forward possible solutions to address societal problems. On the other hand, the ‘politics stream’ examines the contextual drivers (barriers and facilitators) that underpin authorities’ adoption of policy solutions: public opinion, electoral payoffs, power dynamics, etc. A key trait of the MSF is that it was originally developed in a high-income, democratic country (the USA): it focuses on domestic actors as beneficiaries, funders, voters, supporters or promoters of health interventions. Our own experience^15^ is that this is a real strength for a middle-income democratic setting like Ghana, as embracing the full spectrum of domestic actors and acknowledging their respective agency allows for a fuller understanding, less distorted than one that would, for instance, emerge from focusing on the sole donor–government dyad (for a similar view on the need to move beyond this dyad, see Sriram et al).^19^

Our approach for this study in Ghana was to characterise the situation by using the three streams to identify factors impeding the allocation of public resources to mental health. We examined (a) whether mental health conditions are being seen as a societal problem by the Ghanaian society, and if not, the possible reasons for that; (b) the availability of interventions as possible solutions and the existence and dynamism of policy entrepreneurs; and (c) the status of these solutions as political priorities in the political system of Ghana. We also sought to identify potential policy windows or opportunities to finance or prioritise funding for mental health conditions.

## Method

The study was conducted in Ghana across prominent public health actors. Data collection was conducted in two phases. The first was a document analysis, and the second consisted of key informant interviews.

We first reviewed relevant policies, strategies and guidelines on mental health in Ghana, as well as academic papers. We conducted a rapid literature search across appropriate databases and Google Scholar. Local technical experts, including mental health officers at the WHO Country Office in Ghana, have extensive knowledge of the Ghanaian health system; this helped in retrieving relevant reports and evidence to support the analysis. Institution reports were also retrieved. We then developed a data extraction sheet and extracted relevant information on the mental health policy, financing, priority setting, barriers to policy implementation and opportunities for financing mental health.

Semi-structured interviews were conducted among relevant policymakers, managers, researchers, technical assistants, implementers and advocates in Ghana. We also interviewed clinicians across different levels of the health system to understand how policies are translated into service delivery. Participants were purposely sampled for the study. Following ethical approval (Protocol ID# GHS-ERC 007/03/24), letters of permission for the study were drafted and sent to the relevant institutions and stakeholders with support from the WHO Country Office in Ghana. Emails were also sent, and telephone calls were made to relevant key informants.

The interviews were facilitated by an interview guide, developed in line with the MSF. A total of 33 participants were interviewed (Table 1); the interviews lasted about 45 min. We deliberately included relevant stakeholders from different institutions, from the highest level of mental health policymaking to the lowest level of operationalising or implementing these policies. The interviews were conducted between March 2024 and March 2025. All interviews were conducted face to face and, with participants’ consent, were audio-recorded. The research team continued with recruitment and interviews until data saturation was achieved at the 33rd participant. We did not aim to achieve saturation across the different professional ranks; instead, we targeted overall saturation, which was achieved. We also took field notes during the interviews.

**Table 1:** Characteristics of study participants Table 1 long description.

Category	Frequency
Gender	
Male	12
Female	21
Category of participants	
Mental health nurse	2
Community mental health nurse	2
Psychiatrist	2
Family medicine physician	6
Internal medical specialist	3
Development partners	5
Policymakers	3
CSO/non-governmental organisations	4
Patient advocates	1
Health financing expert	2
Researchers	3
Years of professional experience	
3–5	5
6–10	10
11–15	9
16–20	3
20 and above	4

CSO, Civil Society Organisation.

We analysed interview transcripts deductively by developing a coding frame aligned with the MSF’s key domains. To ensure intercoder reliability, two researchers independently extracted the first three documents/reports and used a similar approach to code and analyse the transcripts. This resulted in very high intercoder reliability, minimised errors and bias, and enhanced the overall quality of the evidence.

The triangulation of the three primary data sources (scientific literature, policy documents and semi-structured interviews) enhanced the trustworthiness of the results. For instance, many of the observations shared by respondents on the problem stream have empirical counterparts in the body of scientific studies conducted in Ghana. We assess that the preparation of the study, its conduct and the writing of the paper also benefited from early choices made to secure reflexivity, including the diversity of perspectives within the research team.

Overall, the coding highlighted issues that, as described by stakeholders and in the situation analysis, are significant concerns regarding the financing and prioritisation of mental health. Existing policy attempts to proffer solutions and strategies to tackle the problem, and the existing actions, new policies and political decisions that affected financing or prioritisation of mental health were captured. Opportunities in the form of windows for funding and prioritisation were also noted and reported, including the profiles and actions of policy entrepreneurs. The document review also provided insights into strategies to address mental health’s financing issues.

To ensure transferability and trustworthiness of the findings, the team instituted several measures. First, a well-developed, detailed interview guide and data extraction sheets were designed to ensure consistency and transparency across the data collection sites, the documents extracted, and the overall synthesis. We also maintained an audit trail through the entire research process, ensured maximum variation in participant diversity and held regular debriefing meetings.

The research team adopted several reflexivity practices worth noting. First, this work was jointly undertaken by researchers from both the Global North and the Global South, with a nearly 50:50 split between male and female authors. Researchers comprised senior, mid-level and junior/early career researchers. All the authors have worked, researched or practised in the field of health financing, public health and mental health in low- and middle-income countries (LMICs). The local authors have worked with, encountered or collaborated with some of the interviewees; however, these prior relationships were kept guarded and did not influence or bias their thoughts or actions throughout the study.

## Results

Here, we present the results from the document analysis and the key informant interviews. First, we will provide a general overview of the low priority given to mental health in Ghana. We then investigate its determinants along the problem, policy and politics streams. Where evidence from the document review was applicable, we presented it first and complemented it with evidence from the key informant interviews. Table 1 below illustrates the different stakeholders interviewed for this study.

### Low prioritisation of mental health in Ghana

Ghana has a structural problem of insufficient allocation of public resources to mental health, which constitutes a major barrier to the delivery of quality mental health services.^20^ This has been a matter of attention by several observers and authors for some time.^21^ It has been covered in scientific papers^22,23^ and in policy briefs by think tanks.^23^ For instance, Adobea et al have already alerted us to a significant gap (85%) between the budget and actual expenditure for mental health in Ghana.^24^

Since 2019, the government’s allocation to mental health has improved. However, the allocations have been irregular and erratic. For example, in 2019, the government allocated GHS 3 570 000.00 (USD 666 169.061), and this increased to GHS 5 314 022.49 (USD 927 921.78) in 2020. However, this allocation was reduced to GHS 3 750 000.00 (USD 632 922.074) in 2021.^14^ Despite this allocation in 2021, only GHS 2 704 730.84 (USD 456 502.36) was released, and similarly, an amount of GHS 6 250 000.00 (USD 692 137.32) was allocated, but only GHS 3 014 156.62 (USD 333 586.77) was released in 2022.^14^

This low allocation of funds is against the background of the provision in the Mental Health Act, passed by Parliament in 2012, that makes mental health services free of charge. However, the implementation or operationalisation of this act has faced major setbacks, resulting in budgetary allocations as low as 3% of total national healthcare expenditure in Ghana.^14^ The direct consequences of this state of neglect are medicine shortages, an underprovision of services (which leads to avoidable morbidity and mortality) and high out-of-pocket payments by households with a member living with a mental health disorder, which leads to impoverishment.

One of our key informants put the allocation of resources for mental health as an afterthought:

‘So, we recognise that there are fundamental problems with mental health. These problems were the fact that mental health was seriously underresourced – inadequate resources for mental health, both financial, human and logistical. We also realised that mental health was underprioritised, and that explains the underresourcing. When they are always sharing the budget, mental health is always the last, always an afterthought.’ (Key informant interview (KII), policymaker)

**Fig. 1:**
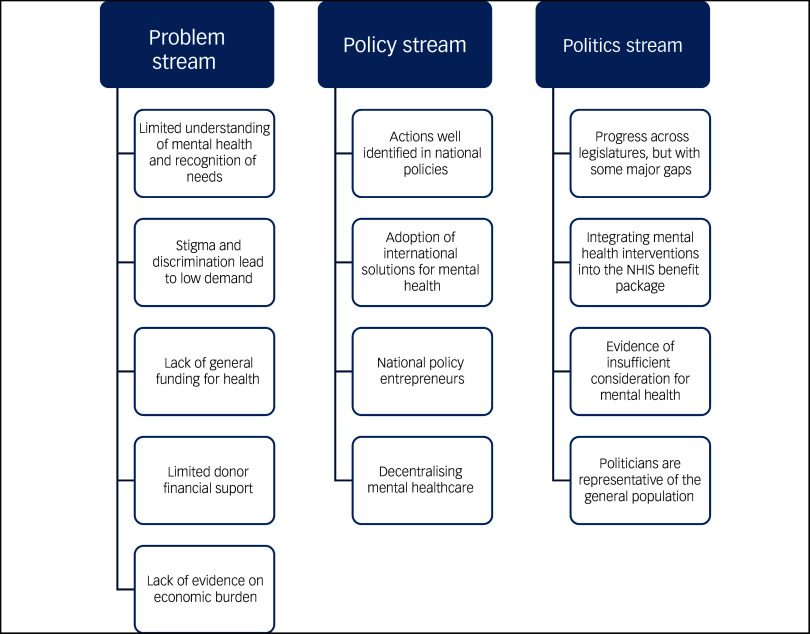
Fig. 1 long description. Prioritisation of mental health as depicted in Kingdon’s multiple stream framework. NHIS, National Health Insurance Scheme.

In the rest of this results section, we examine the causes of this low allocation of public funding. Fig. 1 presents an overview of low prioritisation through the lens of the Kingdon MSF.

### Problem stream

The review of existing documents and evidence from the key informant interviews showed that the problem of mental health financing and prioritisation can be conceptualised in several ways, collectively indicating that mental health is not yet seen as a significant societal issue. This is attributed to (a) misunderstandings of mental health in general society; (b) stigma and discrimination; (c) insufficient funding to the health sector in general; and (d) limited donor interest and (e) catastrophic healthcare expenditure, insufficiently understood or acknowledged as a societal problem.

#### Limited understanding of mental health and recognition of needs

In Ghana, there is still a limited understanding of mental health in the general population. Studies have shown that some people do not understand the risk drivers of mental health disorders, but rather ascribe mental health disorders to other causes, including spiritual.^25–28^ The recent Mental Health Policy highlights the poor understanding of mental health disorders in society as a major problem.^29^

Interviews with the stakeholders also made similar observations. The interviews showed that there is still poor understanding and awareness of mental health, with people attributing mental health conditions to supernatural and religious causes. As a result, the public does not see it as a priority that requires government attention:

‘They don’t see mental health as a priority. Indeed, that is part of it. And there is so much superstition in Ghana. Ghana is a very religious country; I can assure you. Probably about 98% are religious, which is good. The challenge, however, is that we take our religiosity to superstitious dimensions…No young person dies for no reason; there’s nothing like natural causes of death for a young person; somebody, a witch, a wizard, or some evil spirit must be behind. So, we interpret everything in the religious sense; especially issues we don’t understand.’ (KII, policymaker)

#### Stigma and discrimination lead to low demand

Sociocultural beliefs and practices have resulted in high stigma and discrimination, and this is replete in the Ghanaian literature on mental health.^30,31^

The high level of stigma discourages utilisation and sometimes adds an extra layer of workload to the health staff:

‘Most of them don’t want to visit the facility because they don’t want people to know or realise that their relative is suffering from mental health conditions. And sometimes you need to follow them. You need to go home to give them the treatment they need.’ (KII, community mental health nurse)

The prevailing sociocultural and religious beliefs sometimes blame people with mental health disorders by attributing their condition to some evil they have committed. This could divert national attention and, consequently, undermine support for allocating sufficient resources to mental health. This leaves people with mental health disorders alone with the costs:

‘The stigma surrounding mental illness often deters individuals from seeking formal mental health services, leading them to opt for alternative or traditional healers whose services come with out-of-pocket costs. A lack of awareness about mental health, as well as the services available, tends to have the same effect as stigma, driving out-of-pocket spending upwards.’ (KII, CSO mental health advocate)

An interesting twist to this is the fact that policymakers and elites in society may themselves be confronted with mental health challenges within their family. They recognise that mental health is a major issue but are themselves affected by the stigma; this does not encourage them to come out and openly promote larger societal support:

‘But there’s an increasing need. Even with the top people, though they are not giving us adequate attention, now and then, they would call us to report on a child or nephew who is struggling with mental health. Yeah, they call us for support. But they wouldn’t come out in the open because of stigma.’ (KII, policymaker)

#### Lack of general funding for health

Mental health is an ‘orphan problem’ in a sector that itself seems to have lost its precedence in the national policy area. Recently, government allocation to the health sector has been inconsistent and below international commitments. The health budget (as a proportion of the overall government budget) declined from about 12 (2014–2016) to 9% (2017–2020).^32^ A regressive development has been the adoption of the Earmarked Funds Capping and Realignment Act (Act 947) in 2017, which established that the statutory allocation of funds through earmarked taxes might not exceed 25% of total tax revenue.^33^ This substantially affected the funding of the national health insurance scheme, which was expected to be funded by revenue from the National Health Insurance Levy. Furthermore, the Earmarked Funds Capping and Realignment was amended in 2023, reducing the figure from 25 to 17% of tax revenue.^34^ In 2020, Ghana’s current health expenditure was 4% of the gross domestic product (GDP), a figure lower than that of countries in sub-Saharan Africa and LMICs, where current health expenditure is 6.5 and 5.4%, respectively:^35^

‘I have yet to see any bold policy decision by the government to prioritise health, with the necessary investment and funding allocation. In some years, you will instead see a decline in budgetary allocation to health. We need a paradigm shift in the healthcare funding model in this country.’ (KII, health financing specialist)

#### Limited donor financial support

In some countries, external aid compensates for the limited availability of public resources. This is not the case for mental health in Ghana. Unlike other public health issues such as malaria, tuberculosis, HIV and maternal/child health, mental health does not enjoy much support from development partners active in the country.

One of our KIIs reported that, until recently, the Mental Health Authority benefited from a UK Government-supported mental health project. This programme was key to making programmatic progress: because of limited state funding, the mental health authority primarily relies on it to implement its activities:

‘…. and, of course, the mental health authority is keen to secure operational funding. I mean, they rely primarily on donors to function adequately and fund their plans. This is not sustainable, and this is why there is an urgent need to have the mental health levy as a reliable funding source for mental health.’ (KII, mental health programme implementer)

Yet its fate (cut in 2020 because of both reductions and reallocations of UK aid) reveals the fragility of the mental health programme itself. It was reported that the budget was cut by 75%, which affected the completion of specific interventions.

The interviews showed that, despite efforts to raise funding and attention for mental health, mental health is not an area of interest for development partners:

*‘*You need to push and push. You go to somebody, either a local industry or development partner, and they tell you that mental health is not their area of interest. They tell us This is not part of our main programmatic areas.’ (KII, policymaker)

#### The economic burden on households is insufficiently documented

The National Health Insurance Scheme (NHIS) does not compensate for the insufficient government funding and development assistance. Indeed, the NHIS, until recently (2025), did not cover mental health disorders except for other physical health conditions diagnosed among people living with mental health. This left the burden upon households. As highlighted in a recent report by the Ghana Mental Health Authority:

‘In the government hospitals and health centres, mental health services are provided on a cost-sharing basis, that is, patients pay some charge for accessing healthcare while the government provides some subsidy. This covers primary, emergency, outpatient, inpatient, psychotherapy, medication, and substance abuse management services.’^14^

There are indications that mental health is a major source of economic distress for families. More than 10 years ago, Addo and colleagues reported an average household (direct and indirect) cost per month of about USD 60, relative to an estimated average monthly household income of about USD 184.^36^

The issue of high out-of-pocket payments was indeed reported consistently across key informants. First, the high cost of treatment deters frequent visits and consultation. So, the low demand reported above is also attributable to the financial barrier:

‘Due to the high cost of admissions and access to mental healthcare in the major psychiatry hospitals and units, patients together with their caregivers and family members, sometimes avoid seeking care and resort to care with traditional, alternative or spiritualists.’ (KII, CSO mental health advocate)

Out-of-pocket payments also affect health outcomes:

‘A high proportion of patients seeking psychiatric or mental healthcare at these facilities encounter difficulties affording the necessary medications for their treatment. Insufficient availability of psychotropic drugs also contributes to an escalation in relapse rates among individuals receiving mental health treatment. Patients may incur additional costs related to transportation when seeking mental health services, particularly as most need to travel long distances to access specialised care.’ (KII, Mental Health Authority)

Although our key informants were concerned with the economic cost on households, we failed to access rigorous studies documenting the situation of households with a member with mental health disorders. Most studies on out-of-pocket payments in Ghana are either cross-sectional^37^ or too specific.^38^

### Policy solution stream

Here, we attempt to answer whether the low funding for mental health is because of a lack of proposed solutions to address the problem or a lack of policy entrepreneurs. The answer is negative: clear action points have been put forward by various actors and integrated into national laws, policies and strategies. We observe the close connection between these developments and more general diagnoses and propositions made at the international level. We also find evidence of policy entrepreneurs who complement each other in their respective roles and consistently promote the implementation of these solutions.

#### Actions are well identified in national policies

Over the past three decades, Ghana has issued several national strategic documents (see Table 2). They list action points and help to structure the development of a coordinated response to address the mental health burden:

**Table 2:** Key policy development milestones in Ghana

Policy milestones	Year
Mental Health Policy	1994
Mental Health Policy (Revised)	1996
Mental Health Strategic Plan (2008–2011)	2009
Mental Health Act, 2012 (Act 846)	2012
Establishment of the Mental Health Authority	2012
Establishment of the Mental Health Board	2013
Mental Health Strategic Plan (2014–2017)	2014
Mental Health Strategic Plan (2019–2022)	2019
Mental Health Policy (2019–2030)	2018

‘We now have a policy in place, and it also includes pipe-punching. The mental health policy from 2019 to 2030 is also firm. So, once we can push the policy forward and put it in place, we are getting there.’ (KII, policymaker)

The promulgation of the Mental Health Act in 2012^39^ was a key development for the country. The issue, however, is that its implementation has been too incomplete (see further in section ‘Politics stream’). Still, there have been several positive developments. A recent review cites interventions in schools, health centres, traditional healing conventions and at the community level, as well as interventions targeting children and adolescents.^40^

#### Decentralising mental healthcare

A key feature of Ghana’s mental health policy reforms is the effort to decentralise mental healthcare to primary care settings, including support systems at the community level. Although mental healthcare in Ghana is still highly institutionalised, hospital-based care and often urban-centric, there is a policy shift toward expanding care to primary care settings at the community level, as aligned with WHO policy/recommendations. As a result, mental health clinics or psychiatry units are established in most district hospitals:

‘Mental healthcare is shifting from tertiary, hospital-based care to primary and community care. It took us a while to get there and that has always been our position as an NGO [non-governmental organisation] and a CSO for the past two decades, pushing for this to happen. There are many barriers to care, especially at the community level. Now we have what we call the community mental health nurses who are placed at primary care units within facilities to ensure easy access to mental healthcare.’ (KII, CSO mental health advocate)

Infrastructure for mental health has also undergone a significant facelift through service delivery decentralisation. The observation by a policymaker lends credence to this:

‘Since time immemorial, there have existed only three psychiatric hospitals in the entire country, but this is changing and bringing mental healthcare closer to those in the middle and northern belts. A new psychiatric hospital is being built in the middle belt of the country to tackle the geographical barrier to accessing dedicated mental healthcare. Mental health units are now also established in some district hospitals.’ (KII, policymaker)

#### Adoption of international solutions for mental health

These interventions and national-level mental health programme actions, more broadly, have often been informed by and benefited from global propositions such as the WHO’s Comprehensive Mental Health Plan 2013–2030, which has sought to galvanise local and national-level commitments and support for mental health.^41^ In response to the WHO Director General’s 5-year Special Initiative for Mental Health, which sought to ensure access to the highest standard of mental healthcare for everyone in the selected countries, Ghana has closely collaborated with the WHO Country Office. As a result, a national implementation plan has been developed to address access barriers, including tackling and supporting advocacy and human rights among people with mental health disorders. Other moves by the country to adopt international solutions and initiatives include implementing the WHO Mental Health Gap Strategy and integrating it into primary healthcare.^42^ The adoption of the United Nations Convention on the Rights of Persons with Disabilities in Ghana was also a watershed moment that, to some extent, guaranteed the rights of people living with mental health disorders. The enactment of the Mental Health Act in 2012 was also in fulfilment of this Convention.^43^ Also worthy of mention is the impressive rollout of the WHO QualityRights in Ghana, an initiative that sought to address human rights abuses as experienced by persons with mental health disorders.^44^

Some participants expressed satisfaction about the strides made in the adoption and implementation of mental health policies in Ghana:

‘Ghana is among many countries referenced for adopting the WHO Director-General’s 5-year Special Initiative for Mental Health. Aside from this, others have been implemented in the past, including the WHO Mental Health Gap Strategy. I think the implementation has so far brought about some steady progress in improving access to mental healthcare, but we are not there yet. A lot is still outstanding.’ (KII, interventional development partner)

#### National policy entrepreneurs

In Ghana, CSOs, researchers and mental health patient advocates have played a pivotal role in policy development and implementation, including the enactment of laws and legal provisions. Our analysis and interviews showed a vibrant activism, pushing for funding, better policies and active involvement in the design or implementation of concrete solutions to improve access to mental healthcare. Some supported the state agencies in establishing mental health tribunals:

‘We have a very close working relationship with the Mental Health Authority, and we supported them in establishing and piloting the Mental Health Review Tribunal.’ (KII, CSO mental health advocate)

These coalitions and CSOs, through their grassroots-level action, such as the establishment of self-help groups, tap the power of persons with lived experience, including in remote areas:

*‘*We very much prioritise what we call peer and self-help support, using persons with lived experience to use their innate potential and their lived experiences to help each other. So, in a bit, that’s what we have been doing since 2002. Our main areas of operation have been in what we consider the disadvantaged parts of Ghana. And that is the northern, rural locations of Ghana. So, the five northern regions of Ghana. And in the southern part, we have focused on the slum and migrant communities.’ (KII, CSO mental health advocate)

These activities have enabled them to make significant inroads in demonstrating the relevance and feasibility of an alternative vision for mental health service delivery beyond psychiatric institution-based care:

‘I would say, for our work, we have, for almost 20 years now, supported up to 200,000 people benefiting from community-based mental healthcare services, which are within proximity to them. Because our whole approach is that mental healthcare should be de-institutionalised and integrated at the primary care level. So that anybody needing mental healthcare services can access them beyond just setting up institutions like psychiatry facilities. So, we want to see that mental health is part and parcel of general healthcare services.’ (KII, CSO mental health advocate)

These achievements fit well in the broader effort supported by other actors, including ‘organisational policy entrepreneurs’ such as the WHO, and get integrated into the national strategy led by the Mental Health Authority.

### Politics stream

The MSF provides a third area of investigation: would the low public funding for mental health be a result of poor alignment between politics and officials’ political interests and agenda? We have found evidence of ongoing political attention to mental health; however, it has not led to a real financial commitment. Champions of mental health have been navigating the unfavourable context for decades and have recently adopted a strategy to secure mental health interventions into the NHIS benefit package.

#### Progress across legislatures, but with some major gaps

Ghana is a vibrant democracy, with frequent alternation between political parties holding the majority in parliament and in charge of the national government. Governments after governments have demonstrated some level of support for mental health, as seen in Table 2, which shows a continuity of policy development over the past decades. Currently, there is a mental health policy^29^ spanning 2019–2030, developed under the immediate past government. This represents a significant milestone in providing a blueprint for improving access to and mobilising funds to support mental health activities in Ghana.^45,46^ Before this, a different government, following local and international pressure to improve the situation of mental health conditions in the country, enacted the Mental Health Act in 2012 and established the Mental Health Authority and a Board.^47,48^

However, observers see this as a ‘low equilibrium’ as no government has made any significant financial commitment over the years. There has been considerable frustration with the incomplete implementation of the Mental Health Act (Act 846) of 2012. This Act represented a much-needed legal development, replacing a Mental Health Decree adopted in 1972,^49^ and provided the necessary impetus for action in light of the changing mental health situation and the need for new responses aligned with the local needs and expectations of the Ghanaian population. However, there have been significant challenges in implementing the Act. The mental health levy announced in the Act has never been established. It was the institutional solution imagined providing sustainable funding for mental health. Scholars^49^ and our key informants concur that this has been a significant policy deficit:

‘Ghanaian politicians are not committed to mental healthcare in Ghana. For me, it is about the lack of political will and commitment. This is what we have lacked in Ghana over the past decades. No political party or government has been bold enough to trigger the process for the introduction of the mental health levy yet, although the Mental Health Act stipulated the need to introduce such a levy to ensure we have a sustainable means of financing mental health in Ghana.’ (KII, CSO mental health advocate)

#### Integrating mental health interventions into the NHIS benefit package

For years, mental health actors fought to have the Act implemented to advance free access to mental health services for all Ghanaian citizens, one of the Act’s key provisions. Several pieces of evidence have been used, before and after the Act. For instance, some researchers documented that the GDP loss attributable to mental health was huge:

*‘*We have been giving them figures. In 2009, I was part of a team that studied the GDP loss resulting from mental health. They realised that we were losing 7% of our GDP as a result of mental health issues. That should have been enough for them to recognise that this is a problem. We have been pointing out other data to them, for them to recognise, but they just read it and pass it off.’ (KII, policymaker)

After observing the lack of progress in securing domestic resources, mental health champions decided to pursue another strategy.

Actors, including the CSOs, WHO, researchers, the Mental Health Authority, the Ghana Psychology Council, the Christian Health Association of Ghana and other stakeholders have been advocating for the current NHIS to include four primary mental health conditions (depression, bipolar disorder, anxiety and schizophrenia disorders) to the health benefit package of the NHIS.^50^ There is scientific evidence supporting this prioritisation: studies have demonstrated the cost-effectiveness of interventions addressing these conditions.^51^

They have also flagged that such an integration within the benefit package will help address the exceedingly catastrophic expenditure currently being incurred across the country’s psychiatric units and hospitals. The strength of this proposition is to acknowledge better the reality of the health financing system put in place over the past two decades in Ghana. The drawback is that the entitlement will be secured only for the enrollees of the national health insurance. It remains to be seen how the recent declaration by the immediate past government to include the four mental health conditions in the current NHIS will be implemented.^52^

#### Other evidence of insufficient consideration for mental health

The authorities’ low attention also manifests itself in other key political moments. For instance, an analysis of the statutory State of the Nation’s addresses by each sitting president of Ghana from 2007 to 2021 revealed that no mental health disorder was highlighted in any of the addresses during this period.^53^

The management of the brain drain of psychiatrists and other mental health specialists could be considered as another piece of evidence of insufficient action. Although studies have reported this brain drain issue,^54^ it remains off the government’s radar. Medical staff working abroad have also become a product of export, a source of remittances to the government, raising questions about political alignment and interest, and the trade-off often made at the detriment of mental healthcare in the country. As reported by some researchers, limited funding for mental health affects the support available for recruiting and retaining mental health providers, further exacerbating shortages.^55^ Because of these shortages, the few tend to work in multiple roles, from community-based out-patient to in-patient services.^56,57^ One policymaker summarises the situation of the brain drain and the chronic shortage of mental health workers in Ghana as follows:

‘There is a notable deficiency of psychiatrists in Ghana, with only 73 psychiatrists available to serve the entire population. As a result, there is a disproportionate distribution of psychiatrists in Ghana, with 46 of the 73 psychiatrists concentrated in the Greater Accra Region. At the same time, regions such as the Savannah, Oti, North-East, Brong Ahafo, Ahafo and Bono East lack psychiatrists entirely. This makes continuity of care difficult.’ (KII, policymaker)

#### Politicians are representative of the general population

As previously reported, stigma, religious beliefs and wrong representations of mental health are replete in Ghanaian society.^30,31,58^ Those also prevail among politicians. Several informants reported that the culture in Ghanaian society is supportive of (a) competing explanations for mental health problems and (b) competing solutions (traditional medicine, prayer and religion) in line with these non-scientific explanations. These cultural and other contextual issues allow politicians to do little and rather focus on other priorities that are more highly recognised by society.

### Opportunities for domestic financing and prioritisation

Despite financial barriers to allocating more resources to mental health and to pushing this issue onto the agenda of policymakers and stakeholders, during in-depth conversations, stakeholders proposed several strategies to optimise the use of existing resources and improve overall mental healthcare in Ghana. The strategy suggested includes (a) expansion of the health insurance coverage to include mental health services, (b) operationalisation of the Mental Health Levy, (c) government subsidies and local production of psychotropics, and (d) efficient use of existing resources.

#### Opportunity 1: expansion of the health insurance coverage

The current NHIS broadly covers other health conditions of people living with mental health disorders. Still, in the wake of the poor funding situation, it is incumbent on the governments to consider funding more mental health conditions to reduce the out-of-pocket expenditure, as this is a more sustainable measure to address the long-standing neglect of domestic financing for mental health:

‘Overall, it is a good start to include mental health in the NHIS package, which we have been advocating for because the government’s commitment to pay for mental healthcare services is not met – they have always been in arrears. Because of that, when people with mental health conditions go for treatment, they have to pay out of pocket.’ (KII, mental health advocate)

Despite the government’s decision to expand the NHIS package to include four primary mental health conditions, the government has yet to develop operational guidelines for implementation.

#### Opportunity 2: operationalisation of the Mental Health Levy

The Mental Health Act in Ghana enjoins the establishment and operationalisation of a Mental Health Levy, but this has yet to be done. Participants identified this as an excellent opportunity to raise funds and secure a sustainable, earmarked financing source for mental health in the country. To progress this, participants recommended that the government revisit the Act and the provisions for the development and implementation of the Mental Health Levy to create a more sustainable flow of funds:

‘If free care were possible, we would need to establish a mental health fund. With the enactment of the law, a fund has been established, all right, but we need to feed the fund with money – the Mental Health Levy. It’s about time we pushed for the operationalisation of the Mental Health Levy. That is one of the low-hanging sustainable revenue streams we can plug in.’ (KII, policymaker)

Some were of the view that the existing COVID-19 levy should be diverted, and the funds directed to the Mental Health Fund, since the pandemic was now behind us.

#### Opportunity 3: government subsidies and local production of psychotropics

Another strategy suggested by participants as a potential source of sustainable financing for mental health is the need to support and invest in local production of medications and other essential consumables for mental healthcare. They believed that essential medicines for mental health should be produced locally by local pharmaceutical companies. This is a more sustainable path to achieving universal health coverage: it will improve access, minimise imports of psychotropic and other essential medicines for mental health, and free financial resources for different needs:

‘The bulk of the problem lies in medication. And of course, in terms of the psychotropic drugs, some of them are quite archaic. We now have new medicines that have been modernised and improved, but the cost is a problem. So, quite a few patients are unable to afford them. However, with little push and support from the government, a number of the local medicine manufacturing companies can produce these psychotropics locally. This is an opportunity we should not miss. Some may just require some support from the government to procure and import some of the raw materials to do the production locally at a reduced cost for the patients at the end of the day.’ (KII, mental health specialist)

To encourage the production and importation of drugs, the government should subsidise them to ensure reasonable mark-ups. Essential psychotropics should be given a special waiver for importation with tax breaks or a reduction to improve supply in the country:

‘In the absence of immediate policy actions to fund the mental health sector in this country, one immediate strategy will be to ensure the government provides subsidies for medications and essential medical supplies for the treatment and management of mental health conditions. This will help reduce mark-ups by importers and local manufacturers of medicines and supplies, a key driver of the high cost of medications and psychotropic drugs.’ (KII, mental health service provider)

To achieve this, broad stakeholder consultation is needed to review existing medicine pricing policies and strategies, given the volatile nature of the pharmaceutical market landscape.

#### Opportunity 4: efficient use of existing resources for mental health

Although not widely expressed, some stakeholders complained of waste and inefficiencies in the health system. In their view, a more efficient use of the limited resources in the health systems is imperative. In an editorial, Agorinya and colleagues^54^ made a poignant argument for harnessing local resources and using them prudently. This point was made to support the idea that limited resources exist, yet the little that is available is not used to the best effect:

‘The default argument has always been the lack of funds and the need for more funding to solve the problem, but we can still make a difference in mental healthcare in Ghana with the limited resources available. We need to build systems, institutions and capacity to ensure we put better use to what we already have. I believe we can achieve a lot if we adopt strategies to block leakages, ensure transparency and accountability.’ (KII, mental health specialist)

## Discussion

### Summary of findings

This study set out to qualitatively evaluate domestic financing and prioritising for mental health in Ghana, exploring the barriers, potential opportunities and recommended strategies to increase policy attention to mental health. The analysis of published literature and the key informant interviews revealed that, despite the burden of mental health in Ghana, mental health is not yet seen as a societal priority by the population or their political representatives, at least not sufficiently to warrant the necessary financial commitment. The constraints to progress are not at the level of diagnoses, plans, solutions or actors defending them: Ghana has the necessary national policies, is keen to adopt international solutions, has the legislative framework and an ecosystem of policy entrepreneurs. This indicates that mental health champions, including persons with lived experience, will still have to be proactive to align current mental health problems with political interests and agendas, changing governments and each government’s new priorities.

### Findings compared with the literature in other countries

The low prioritisation situation that we report in Ghana is not unique: mental health has not enjoyed high prioritisation in many countries.^8,59^ Mental health shares the fate of NCDs,^60,61^ despite their increasing burden in LMICs. The low funding and prioritisation for mental health calls for more empirical research on the underlying drivers. In our sister study on diabetes and NCD,^15^ we reported how a coalition of actors generated and used local evidence to convince policymakers and legislators of the need for health taxes.^62^ In agenda setting and policy prioritisation for mental health, the use of evidence is critical. Brooks et al recommend that this evidence be multidimensional, involve several stakeholders and actors, and address stigma.^63^ As we observed, the stigmatisation of mental health is indeed rife in Ghana. It often leads prominent people in society, including policymakers, to distance themselves from the conditions or to have reason to underprioritise it in policy making and resource allocation.

One of the dimensions to be documented is the economic burden on households with a member with a mental health condition. Today, there is global recognition that healthcare expenditures are a significant cause of financial hardship and poverty among families – this is one of the core metrics of universal health coverage. As reported in our study, stakeholders stressed that the lack of funding and its prioritisation for mental health contribute to the high out-of-pocket payments by Ghanaian families. Studies conducted in other settings have reported that the cost of mental healthcare affects individuals and their families, resulting in high out-of-pocket payments and catastrophic health expenditures.^64–66^ We have reported that such studies were missing in Ghana – this is a handicap for powerful advocacy.^67^ It will also be essential to reflect on the implications of out-of-pocket payments for health financing policies and other interventions aimed at achieving universal health coverage. A key issue will be to ensure access for all (not only for those enrolled in the national health insurance).

As in other contexts,^68–70^ our study found that, at the societal level, multiple interpretations and attributable causes of mental health conditions exist. Some attribute mental health issues to spiritual, religious and cultural causes, and not neurological causes. Studies in Ghana and other contexts are replete with this phenomenon.^27,71^ Both the traditional culture and some Christian churches promote other aetiologies of mental health problems. The adoption of the MSF reminds us that this has direct implications: alternative interpretations that shift the blame away from natural causes legitimise other solutions, such as traditional medicine and prayers. Such interpretations and stances may lend credence to the view that mental health is not primarily a governmental responsibility, but a private one, and that individuals can provide the solutions, commitment or the obligation to act. This also tends to give politicians some legitimacy or even conviction not to prioritise mental health. It may also create extra hurdles for the most committed policy makers who may wish to act but are confronted with the religious and cultural dimensions of solutions to the problem, thus compromising efforts to prioritise resources for mental health.

### Directions for action

The study shows that Ghana is making progress toward policy change. The recent integration of interventions for four mental health conditions into the national health insurance is a positive development.^50^ This was achieved through intense advocacy and lobbying, as well as incessant engagement with legislative and executive bodies. A key test for the politics stream is whether the democratic alternation resulting from the December 2024 election will affect the implementation of this decision. There are other reasons for hope: the steady expansion of infrastructure, the ongoing development of mental health capacity in primary care facilities, the development of different cadres and specialisation in psychiatry are all other indications of increased attention and support for mental health, including among the new generations of health workers.

There is potential to expand the coalition of change. As identified by the Mental Health Leadership and Advocacy Program team, advocacy and grassroots movements will remain pivotal in pushing for the transformation of policies to prioritise and finance mental health interventions in Africa.^72^ Given the overall low recognition of mental health as a societal problem, and thus, the low prioritisation by policy makers, it will take massive awareness creation and education drives, grassroots movements and advocacy to change the status quo.^72^ Clinicians have a key educational role to play at both the facility and community levels, among others, in promoting the availability of solutions and combating stigma. Mental health education and advocacy should be inclusive, involving families, caregivers and individuals living with the conditions. Getting policymakers and politicians to prioritise mental health needs and concerns requires departing from the often top-down approach practised in public health.^73,74^ Supporting the emerging ecosystem of CSOs, academics, state actors and activists in the mental health front in Ghana, through financial resources, learning opportunities and frameworks, and interventions should be a priority.^72,75^

A core objective should be to increase government spending on health, specifically on mental health, to close the huge treatment gap.^76^ Recent health reforms include the inclusion of four primary mental health conditions in national health insurance coverage. This is a key test for the government of Ghana. These and other solutions are reported in Table 3, which highlights contextual barriers to financing and prioritising mental health, as well as proposed solutions.

**Table 3:** Contextual barriers and strategies/solutions to mental health financing and prioritisation Table 3 long description.

Contextual barriers	Policy solutions
Limited understanding of mental health and recognition of needs by the society Stigma and discrimination lead to low demand Lack of general funding for health Limited donor financial support Insufficient documentation of economic burden on households	Broad coalition of change Expansion of the health insurance coverage Operationalisation of the Mental Health Levy Government subsidies and local production of psychotropics Efficient use of existing resources for mental health

The point must be emphasised that although calls for increased funding are critical and necessary, further calls to ensure the efficient use of available resources remain paramount. Adopting low-cost interventions, such as the WHO Mental Health Gap Action Programme, and instituting appropriate governance and monitoring systems to ensure the prudent use of available resources is also paramount. All in all, mental health remains high among the Ghanaian populace, with evidence showing a very high treatment gap and a high prevalence of about 10%,^77,78^ whereas those with severe forms of mental illness are estimated to be up to 3%.^77,78^ These figures are relatively higher than those in high-income countries.^79,80^ The findings highlight the financing and political neglect of mental health in LMICs. All hands must be on deck to explore opportunities, including local/domestic funding, private-sector funding and, above all, the prudent and efficient use of existing resources. These may involve reforms to existing funding policies and schemes and ploughing more resources into mental health conditions.

### Study limitations and ways forward

Although this study offers important insights for policy and practice, it has some limitations that must be acknowledged.

Our experience with this study and the sister study on NCDs^15^ confirmed the relevance of considering a broad spectrum of societal actors to document the three streams of the MSF. The problem stream might be the most challenging, given that ideally, one should measure the views of the whole society. For this study in Ghana, we were able to tap a substantial body of literature. In countries where empirical studies are missing, well-funded researchers may want to consider a poll. Interviewing people with lived experiences of mental illness or their caregivers would undoubtedly have enriched our understanding. On the policy stream, we would also encourage researchers to look for private citizens acting as policy entrepreneurs; they play a role for some mental health conditions (for example, with autism in the USA).^17^

Additional quantitative data on fiscal trends and mental health spending could have further enhanced the rigour of the findings. The paucity of financial data is not specific to Ghana.^11^ As argued in the paper, generating evidence on out-of-pocket payment for mental health is also key for advocacy: it proves the distress caused by the insufficiency of public financing.

Trying to identify the reasons behind the occurrence or not of a specific policy development will always have some speculative nature, given the lack of counterfactuals. One way for the scientific community to address this constraint might be to multiply similar MSF case studies on mental health (non-)prioritisation in other countries. Reports of successful mental health prioritisation would be invaluable for identifying enabling factors. Comparing relative successes in terms of prioritisation across health conditions could also be insightful. Finally, in the near future, there could also be opportunities for more quantitative investigation. For instance, the recent brutal decline in international health aid might provide some natural experiment and through the reallocation of domestic resources, reveal ‘authentic’ governmental preferences in some countries. The increasing number of countries institutionalising health technology assessment processes^81^ will also generate new data on prioritisation decisions, which could maybe help to evidence and explain the low prioritisation of mental health interventions. Combining cross-country qualitative and quantitative analyses looks like a powerful way forward.

To summarise, the findings presented here raise important questions for policy action to ensure adequate resources are generated locally and issues of mental health are prioritised. As noted in this study, mental health is not recognised as a significant societal issue, which calls for greater support and investment in raising public awareness through campaigns and activities on mental health, its determinants and the need to seek treatment in a health facility. This will increase demand for services but also put pressure on political parties and the government. Funding for preventive and curative interventions is the obvious other priority. Those must also be duly implemented and enforced. This is a significant policy gap that could be addressed by funding supervision of the plan and project implementation. This is important because the implementation of existing policy solutions is sometimes halted mid-stream or poorly implemented. Such monitoring will help match this. Providing catalytic funding to support evidence generation is imperative. As noted in the findings, there is still limited local evidence on policy prioritisation of mental health and the economic burden of mental health conditions. Such a fund would support evidence generation, providing the impetus for policy interest and investment in mental health to attenuate high out-of-pocket payments.

## Data Availability

All relevant data and materials supporting the findings of this manuscript are reported in this paper. Additional information, if needed, can be obtained by contacting the corresponding author, B.M.

## Table 1 Long description

The table presents the characteristics of study participants, detailing gender distribution, categories of participants, and years of professional experience. The table has 12 rows and 2 columns. The first column lists categories such as gender, category of participants, and years of professional experience. The second column shows the frequency of each category. For gender, there are 12 males and 21 females. The category of participants includes roles like mental health nurse, community mental health nurse, psychiatrist, family medicine physician, internal medical specialist, development partners, policy makers, CSO/non-governmental organizations, patient advocates, health financing expert, and researchers. The years of professional experience are categorized into 3-5, 6-10, 11-15, 16-20, and 20 and above. Notable trends include a higher frequency of female participants and a significant number of participants with 6-10 years of experience.

Navigate back to Table 1.

## Fig. 1 Long description

The diagram is divided into three columns: Problem stream, Policy stream, and Politics stream. The Problem stream includes limited understanding of mental health, stigma and discrimination, lack of general funding, limited donor financial support, and lack of evidence on economic burden. The Policy stream highlights actions well identified in national policies, adoption of international solutions, national policy entrepreneurs, and decentralizing mental healthcare. The Politics stream mentions progress across legislatures with some major gaps, integrating mental health interventions into the NHIS benefit package, evidence of insufficient consideration for mental health, and politicians representing the general population.

Navigate back to Fig. 1.

## Table 3 Long description

A table with two columns and six rows. The first column lists contextual barriers to mental health financing and prioritization, including limited understanding of mental health needs, stigma and discrimination, lack of general funding for health, limited donor financial support, and insufficient documentation of economic burden on households. The second column lists policy solutions to these barriers, such as forming a broad coalition of change, expanding health insurance coverage, operationalizing the Mental Health Levy, providing government subsidies and local production of psychotropics, and efficiently using existing resources for mental health.

Navigate back to Table 3.
